# Cancer therapy-induced cardiotoxicity: mechanisms and mitigations

**DOI:** 10.1007/s10741-025-10531-0

**Published:** 2025-06-07

**Authors:** Solanki Shil, Prabodh Kumar, Kamalesh Dattaram Mumbrekar

**Affiliations:** 1 Department of Radiation Biology and Toxicology, Manipal School of Life Sciences, Manipal Academy of Higher Education, Manipal, Karnataka India 576104; 2 Department of Cell and Molecular Biology, Manipal School of Life Sciences, Manipal Academy of Higher Education, Manipal, Karnataka India 576104

**Keywords:** Chemotherapy, Targeted therapy, Radiotherapy, Cardiotoxicity, Cardiomyopathy, Normal tissue toxicity

## Abstract

Cancer treatments like chemotherapy, radiotherapy, and combined immunotherapies have significantly increased patient survival. However, these treatments are frequently linked to cardiovascular toxicity, which has a significant impact on clinical outcomes and patient well-being. Chemotherapy, targeted therapy, and radiotherapy induce significant cellular stress in cardiomyocytes and endothelial cells, causing DNA damage, activating pro-inflammatory and pro-apoptotic signalling pathways. Cumulative damage causes cardiomyocyte loss, followed by fibrosis, resulting in pathological structural and functional remodelling of the myocardium. Endothelial cell damage disrupts vascular integrity, increasing the risk of atherosclerosis, coronary artery disease, and ischaemia. Over time, these changes can lead to clinical conditions like dilated and restrictive cardiomyopathy, which are frequently accompanied by arrhythmias and can result in heart failure and sudden cardiac death. To overcome this problem, the novel field of cardio-oncology aims to provide effective cancer treatments with a multifaceted cardioprotection approach involving pharmacological, diagnostic, natural compounds, and lifestyle interventions during and after cancer therapy. In this review, we cover the important cancer therapies, and their cardiotoxic mechanisms and detail different cardioprotective strategies aimed at mitigating these adverse effects and improve patient outcomes.

## Introduction

Chemotherapy, immunotherapy, targeted therapy, and radiotherapy are the cornerstones of cancer treatment. Long-term cancer survivors are inevitably harmed by cancer therapy-related toxicity, which includes organ dysfunction, impaired cognitive function, and physiological disabilities that reduce the quality of life [1]. Long-term studies of childhood cancer survivors have shed light on the critical issue of cancer therapy-related cardiovascular toxicity (CTR-CVT) [2]. The childhood cancer survivors study revealed an 8.2 fold greater risk of mortality from cardiac issues when compared to individuals of the same age and gender almost two decades after diagnosis [3]. Similarly, a 15 fold rise in cases of congestive heart failure and a 10 fold increase in other cardiovascular diseases (CVD) were observed among the 14,000 survivors [4]. Hence, to improve survival rates, it is crucial to understand and address these potential challenges associated with emerging cancer therapies.

Despite significant progress in understanding CTR-CVT, several challenges remain. First, there is no universally accepted definition of the condition; it is often clinically defined as a reduction in left ventricular ejection fraction (LVEF). Second, a widely referenced classification system proposed by Ewer et al. [5] distinguishes cancer treatment drugs into two major categories based on their mechanisms of inducing cardiotoxicity: Type I, characterised by irreversible myocardial damage, and Type II, typically causing transient myocardial dysfunction. However, there is a limitation in this classification system as several drugs, like trastuzumab (type II drug), can trigger irreversible myocardial damage, given the treatment is not terminated as and when cardiac complications arise. This highlights the need for simultaneously monitoring the patients for any evidence of cardiotoxic occurrence [6]. This overlap complicates clinical management and reveals gaps in our understanding of the cardiotoxic mechanisms of drugs generally used in cancer treatment [7, 8].

Finally, the pathological mechanisms underlying CTR-CVT remain incompletely understood, making this condition one of the most underappreciated sequelae of cancer treatment [2]. The CTR-CVT can arise due to several aetiologies. Although effective against tumour cells, anticancer therapies trigger a cascade of reactions that damage cardiomyocytes and neighbouring endothelial cells. This can be due to the increased oxidative stress and overproduction of pro-inflammatory and pro-apoptotic cytokines [9]. Cardiomyopathy is an extreme condition caused by the pathological remodelling of the myocardium due to the loss of cardiomyocytes, which leads to heart failure, arrhythmias, and sudden cardiac death [10]. In addition, the degree of toxicities also varies across treatment modalities; with radio therapy, outcomes are influenced by factors such as the mean heart dose and bifurcated radiotherapy schedules. Similarly, chemotherapy-induced toxicities are modulated by drug type, patient demographics, and comorbidities [11].

To address these challenges, the multidisciplinary field of cardio-oncology consisting of experts from disparate fields like cardiology, haematology, and oncology seeks to balance effective cancer treatment while minimising cardiovascular toxicity and avoiding unnecessary treatment interruptions [12, 13]. The 2022 European Society of Cardiology (ESC) guidelines on cardio-oncology emphasise the importance of tailoring cardiovascular care to the individual needs of cancer patients, demanding a multidisciplinary approach to develop personalised strategies for managing cardiovascular health throughout the treatment regimen [14]. Several cardioprotective strategies, including pharmacological interventions, natural compounds and physical exercises during or after cancer therapy, show promise in mitigating late cardiac complications [10]. However, significant gaps remain in understanding the mechanisms driving therapy-induced cardiac damage and the development of universal cardioprotective guidelines. This review examines various cancer treatment modalities and their cardiotoxic mechanisms in cardiomyocytes and endothelial cells. We also discuss various cardioprotective strategies used for mitigating CTR-CVT.

## Cancer therapy-induced cardiotoxicity

Conventional cancer therapy and recently introduced targeted therapies have tremendously improved the lives of cancer patients [15]. Several preclinical models have been considered to understand CTR-CVT because they allow for examining the complex interaction between treatment agents, their cardiotoxic response, and the underlying mechanisms. Once an appropriate model is established, defining a reliable or reproducible endpoint for evaluating cardiovascular toxicity is crucial. A summary of the occurrence and underlying mechanisms of cardiotoxicity associated with various cancer therapies is presented in Table 1.

**Table 1:** The incidence and mechanisms of cardiotoxicity for various cancer therapies. (reactive oxygen species- ROS, chronic heart failure- CHF, tyrosine kinase inhibitors- TKIs, immune checkpoint inhibitors- ICIs)

Therapy type	Incidence	Mechanisms of Cardiotoxicity	References
Anthracycline	5–48% (dose-dependent)	ROS production, mitochondrial dysfunction, DNA damage, and activation of pro-apoptotic pathway leading to cardiomyocyte death	[16–20]
Fluoropyrimidines	1–19%	Coronary vasospasm, ischemic heart disease, and myocardial infarction; linked to *DYPD* genetic variants affecting drug metabolism	[21–24]
Taxanes	5–20%	Oxidative stress, arterial stiffness, and reduced ventricular function; higher risk when combined with anthracyclines	[25–27]
Alkylation Agents	Cisplatin: 6–30% Cyclophosphamide: 7–28%	Apoptosis, inflammation, mitochondrial damage, and impaired endothelial cell function	[28, 29]
Monoclonal antibodies	CHF: 2–7% (early stage BC); up to 28% with anthracyclines	NRG-1/ERBB2 pathway inhibition, oxidative stress, and disrupted cardiomyocyte survival signalling	[30–33]
TKIs	Hypertension: 43%; CHF: ~ 8%	Endothelial dysfunction, NO depletion, mitochondrial apoptosis, and microvascular rarefaction	[34–38]
ICIs	Myocarditis: 0.1–1.3%; Severe: 0.5%	T-cell mediated myocarditis, increased inflammatory cytokines, and immune system hyperactivation	[39–41]
Radiotherapy	Late toxicity > 10%	DNA damage, inflammation, oxidative stress, fibrosis, and microvascular injury; dose and site-dependent	[42–45]

### In vitro models

HL-1 cardiomyocyte cell lines from mouse atria were used to study doxorubicin (DOX) induced reactive oxygen species (ROS) production, oxidative stress, and changes in myocardial energy metabolism [46, 47]. Long-term cultured adult rat cardiomyocytes revealed severe effects of DOX on protein degradation pathways. Higher doses of DOX triggered autophagy, apoptosis, and necrosis [48]. DOX-mediated dose-dependent cytotoxicity was studied in three-dimensional cardiac spheroids created by co-culturing stem cell-derived cardiomyocytes, endothelial cells, and fibroblasts [49]. Cardiac muscle tissue models were engineered from rats and humans to understand apoptosis, contractility, and mitochondrial dysfunction in sunitinib-induced cardiotoxicity [50].

Human induced pluripotent stem cell-derived cardiomyocytes (hiPSC-CMs) offer a promising in vitro model to study drug-induced cardiac dysfunction in arrhythmia modelling, CRISPR/Cas9 applications, and 3D cultures for advanced electrophysiological analysis [51, 52]. The hiPSCs can accurately replicate human cardiomyocytes and offer a more reliable and physiologically relevant tool [53–56]. The hiPSC-CMs were generated from patients undergoing chemotherapy and targeted therapy to understand cardiotoxic effects. Studies revealed severe cardiac dysfunction in patients treated with HER2-targeted drugs, while anthracyclines caused decreased antioxidant activity and increased ROS levels [57, 58].

### Preclinical models

Echocardiographic monitoring of LVEF is commonly used to monitor cardiac function; however, a novel echocardiographic strain imaging technique was developed to detect early cardiac damage more effectively and precisely in mice [59]. ErbB2-deficient mice in ventricular cardiomyocytes developed dilated cardiomyopathy (DCM) [60]. Sunitinib-treated mice showed depletion in coronary microvascular pericytes, leading to cardiotoxicity [61]. Human-like variability in anthracycline cardiotoxicity can be effectively modelled using Collaborative Cross mouse strains [62]. Large animals like pigs were utilised to study early markers of anthracycline-induced cardiotoxicity through advanced imaging techniques such as cardiac magnetic resonance imaging [63]. Proteomic analysis in Zebrafish models has established that DOX induces small molecules, resulting in myocardial damage [64]. Some limitations of animal models are that genetic variability within the same species can influence the study results, and they fail to replicate specific human cardiac responses.

## Chemotherapy

### Anthracyclines

Anthracyclines are the foundation of chemotherapy regimens. Drugs such as DOX and daunorubicin have been successful in treating both solid and haematological cancer types for more than five decades. Although anthracycline treatment has shown better survival rates, one of the significant clinical limitations is the cardiac complications observed in exposed patients [65]. Maintaining a low incidence rate of cardiotoxicity is crucial to minimise the chances of mortality and maximise patient survival. At a cumulative dose of 550 mg/m^2^, the incidence rate is estimated to be as low as 5%. However, for a higher dose of 700 mg/m2, the incidence rate ranges from 18 to 48% [16, 17]. Since anthracycline poses cardiotoxicity, 400–450 mg/m2 has been considered the highest clinical allowance [16]. Older patients receiving a cumulative dose of 400 mg/m2 are more vulnerable to chronic heart conditions post anthracycline treatment compared to their middle-aged counterparts [17]. A cohort study of more than 14,000 childhood malignancy survivors revealed a 2 fold increase in the incidence of cardiac dysfunction in individuals who received a cumulative dose of > 250 mg/m2 [11]. A study evaluated left ventricular function in 38 children with leukaemia undergoing anthracycline treatment. Among them, DCM occurred in 3 patients, while 28.9% showed strain abnormalities in cardiac muscle. The risk of these complications was higher in children with anthracycline doses of more than 240 mg/m2 or who underwent radiotherapy [66]. Anthracycline-mediated cardiotoxicity often leads to the presentation of systolic dysfunction secondary to a DCM phenotype. These abnormalities are observed in 60% of cancer survivors, especially older patients who present with reduced LVEF and ventricular dilation [67].

### Fluoropyrimidines

This is the third most used chemotherapy drug for patients with solid tumours such as adenocarcinoma. For the past five decades, fluoropyrimidines such as 5-fluorouracil (FU) and its prodrug, capecitabine, are associated with significant cardiac complications. The rate of occurrence of cardiotoxicity is between 1 and 19%, and the survival rate of patients experiencing cardiotoxicity is reported to be 1.6–10.2%. Long-term patient exposure can cause angina, arrhythmias, and myocardial infarction [21]. Despite extensive clinical observation, there is a notable lack of consistent risk factors for predicting fluoropyrimidine-induced cardiotoxicity. Some factors that appear to increase susceptibility include combination chemotherapy regimens, a history of pre-existing cardiac conditions, and advanced age [68]. For instance, in a retrospective study comparing the cardiotoxicity between 5-FU and capecitabine, the incidence rates were 4% and 5%, respectively [69]. In a survey of 644 patients who received 5-FU and capecitabine, a total of 4% developed cardiac abnormalities, and 5-FU was associated with a greater risk of cardiotoxicity than capecitabine (10% vs 0.8–4%) [70]. Symptoms such as coronary vasospasm, ischemic heart disease, and myocardial infarction were observed in 4.5% of the 177 patients receiving 5-FU in a case–control study [22]. Emerging evidence suggests that genetic predisposition and clinical factors may play a role in fluoropyrimidine-induced cardiotoxicity. Mutations that increase enzyme activity may lower treatment effectiveness, while those that decrease enzyme activity can boost treatment response but raise the risk of side effects. Deficiencies in the enzyme dihydropyrimidine dehydrogenase (DPD), caused by variations in the *DPYD* gene, result in impaired drug metabolism [23, 71]. A study of 132 patients treated with fluoropyridines identified the *DPYD* c.2194G > A variant as being strongly associated with adverse drug risks, particularly relating to cardiotoxic, gastrointestinal and haematological toxicities [24]. A case report stated the role of thymidylate synthase gene variants in influencing severe cardiotoxic effects in a patient with fluoropyrimidine treatment [72]. Therefore, incorporating preventive genetic testing and integrating pre-treatment protocols could allow for dose adjustments or alternative therapies, potentially reducing adverse outcomes. Current data are insufficient to establish clear criteria linking cardiovascular risk factors to fluoropyrimidine-related cardiotoxicity for identifying high-risk patients, making it difficult to recommend discontinuation of fluoropyrimidine therapy based solely on cardiovascular risk [73].

### Taxanes

Taxanes such as taxol or paclitaxel are used to treat patients with solid cancer types such as ovarian, breast, and prostate cancer, but the clinical significance of taxanes is limited by their cardiotoxicity [74, 75]. Serious cardiac complications, including cardiac ischemia, left ventricular dysfunction, and tachycardia, have been observed with an incidence rate of 5–20% in patients treated with this class of drugs [25, 26]. Increased arterial stiffness and oxidative stress are identified in women with breast cancer (BC) treated with taxanes [76]. In a study of thirty women with BC in which DOX was combined with paclitaxel, an estimated 50% of patients developed left ventricular dysfunction, and 20% developed chronic heart failure (CHF) [27]. In recent decades, cardiotoxicities associated with docetaxel have been reported at an incidence rate of 2.3–8% [77, 78]. However, limited data are available concerning the cardiotoxicity of docetaxel and its combination with DOX for treating patients with solid cancer types.

### Alkylating agents

Cisplatin and cyclophosphamide treat lymphoma, myeloma, Hodgkin’s disease, and sarcomas. Cisplatin-induced cardiotoxicity is observed in patients at a rate of 6–30% [28]. Cyclophosphamide, a crucial alkylating agent, has shown evidence of dose-related cardiotoxicity in the 7–28% at a 180 mg/kg dose. The notable causes associated with cyclophosphamide induced cardiotoxicity are apoptosis, inflammation, and mitochondrial damage, leading to cardiomyocyte damage [29].

## Targeted therapy

### Monoclonal antibodies

Antibodies such as trastuzumab, which are used to treat HER-2 positive BC, stomach, and prostate cancer, inhibit the dimerisation of HER2 receptors and are known to cause several cardiac complications like hypertension and CHF. CHF incidence rates range between 2 and 7% in early-stage BC patients and increase to 2.8% when targeted therapy is combined with anthracyclines [30]. Analysis of BC patients over 2 years showed 4.22% higher chances of CHF when treated with adjuvant trastuzumab therapy [79]. Trastuzumab therapy is associated with a 4–5% absolute risk increase in CHF over 2 years in adjuvant therapy settings. A 2-year analysis reported a 4.33% higher CHF risk in trastuzumab-treated BC patients [31]. Pertuzumab in combination with trastuzumab and chemotherapy for HER2-positive cancer, nearly doubles the risk of CHF (risk ratio: 1.97) however, it does not significantly increase the risk of asymptomatic or minimally symptomatic left ventricular systolic dysfunction [80].

### Tyrosine kinase inhibitors (TKIs)

TKIs such as imatinib, sorafenib (SOR), sunitinib, and bevacizumab are used to treat non-small cell lung cancer (NSCLC), chronic lymphocytic leukaemia, and melanoma. It prevents the binding of tyrosine kinases to its receptors, inhibiting the phosphorylation of key substrates in the cells [81]. TKIs cause a variety of toxic effects, including irregular heart rhythms such as an extended QT interval, high blood pressure, and systolic dysfunction. Clinical trials found that < 50% of TKI patients experienced a decline in LVEF of < 10%, followed by MI, atrial fibrillation, and CHF [34, 35]. Cardiac complications of bevacizumab treatment, such as hypertension, were reported in more than 45% of clinical trials, and when combined with drugs such as 5-FU, irinotecan, and leucovorin, the effects are increased 8 fold [82]. SOR and sunitinib are widely used oral drugs for hepatocellular carcinoma and renal carcinoma and they may induce cardiotoxicity in long-term survivors of these cancers [83, 84]. Among gastrointestinal stromal tumour patients treated with repeating cycles of sunitinib in phase I/II trial, 11% had cardiovascular events, with CHF reported in 8% of patients [36]. In another study, hypertension was observed in up to 43% of cases, while CHF is a long-term side effect showing probable late cardiotoxicity [85]. Further research is needed to identify the underlying pathological mechanisms and long-term adverse impact on cardiomyocytes that contribute to cardiac toxicity with TKI treatment.

### Immune checkpoint inhibitors (ICIs)

Cancer immunotherapy has emerged as an effective cancer treatment in recent years. Monoclonal antibodies such as ipilimumab, durvalumab, and nivolumab target cytotoxic T lymphocytes associated antigen 4 (CTLA-4) and programmed cell death 1 (PD1) and its ligand (PDL1) to increase T-cell activity in cancer treatment [86]. Nearly 40% of patients treated with combined ipilimumab and nivolumab developed immune-related cardiac complications [87]. Several cases of myocarditis and CHF are reported in patients receiving ICIs in recent years, either as a single treatment or in combination with other drugs [88–91]. Clinical trials indicate that the incidence of ICIs-induced myocarditis ranges from 0.1 to 1.3%, with severe cases accounting for up to 0.5% and a high mortality rate in untreated patients. Myocarditis is notably more prevalent in dual checkpoint blockade regimens, such as anti-PD combined with anti-CTLA-4 therapy [39, 40]. Of the 30 cases of solid cancer types like bladder and colorectal cancer, ICIs-induced cardiotoxicity, 25–50% are due to anti-PD-1/anti-PDL-1 inhibitors [92]. In contrast, anti-PDL1 inhibitor cemiplimab has shown no cardiotoxic effects [93, 94].

Certain anticancer drugs are known to be more cardiotoxic than others. Factors such as the duration and dose of the treatment and the type and stage of the cancer also influence CTR-CVT. The incidence rate of cardiac toxicity among anthracyclines, TKIs, and proteasome inhibitors is as high as 20–40% [95]. The combination of anthracycline and radiotherapy poses a 15 fold more significant risk of heart failure [96]. In a 5-year follow-up study of non-Hodgkin lymphoma survivors, the incidence of heart failure was recorded at 17% while undergoing anthracycline treatment [97]. Although cancer treatment-induced cardiotoxicity is fatal, in a few cases, the deaths are driven by non-cardiac causes. This may be due to poor outcomes related to fatal cancer types and delays in treatment caused by the manifestation of cardiotoxicity at the time of therapy even at a low-dose administration [76, 98].

## Chemotherapy-induced cardiotoxicity mechanisms

**Fig. 1:**
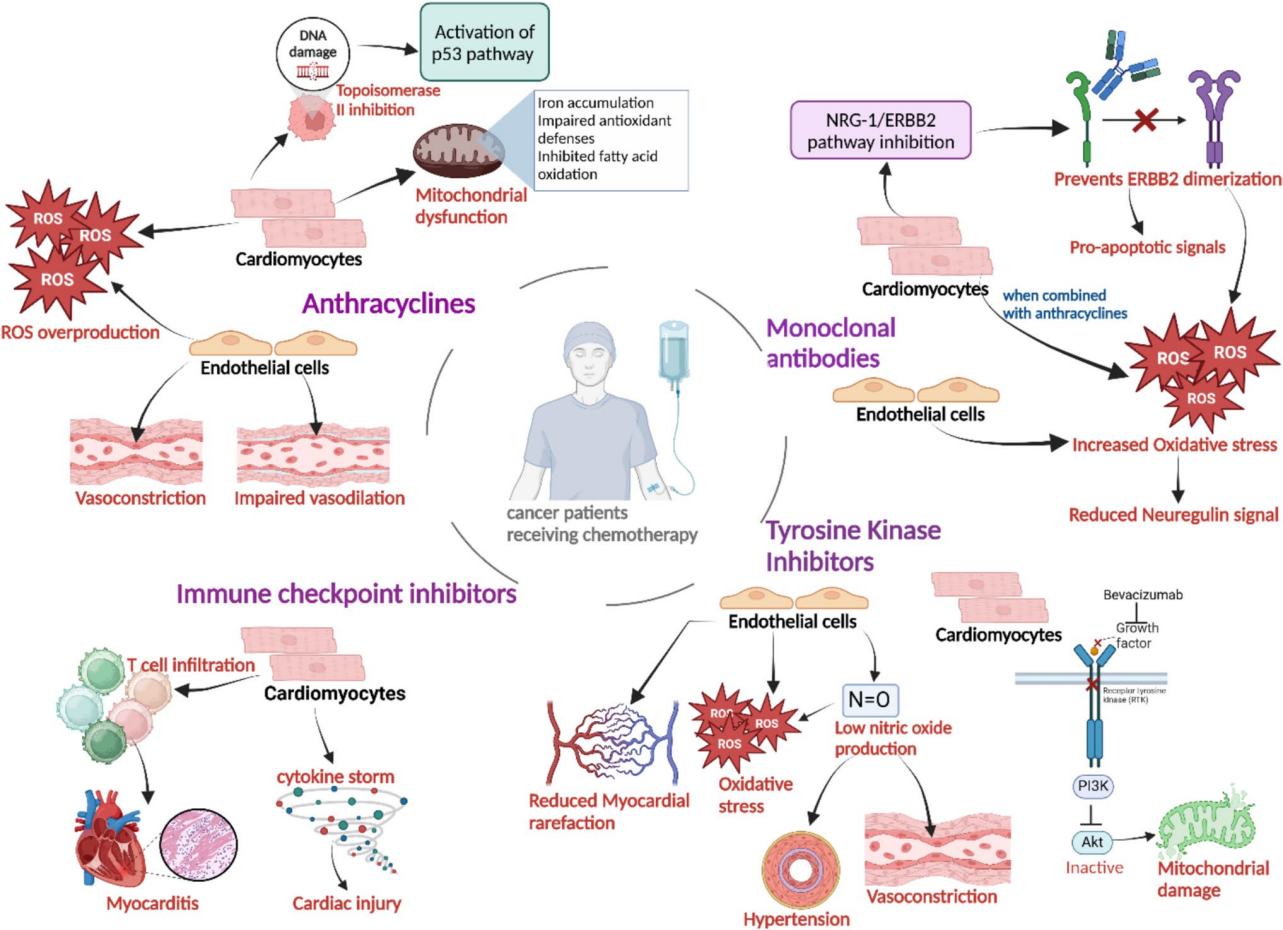
Cancer therapies and cancer therapy-related cardiovascular toxicity. Anthracyclines induce cardiotoxicity via increased ROS production, topoisomerase II poisoning, and mitochondrial disruption. Monoclonal antibodies disrupts NRG-1/ERBB2 signalling, inducing oxidative stress and apoptotic pathways, especially when combined with anthracyclines. TKIs impair endothelial NO synthase activity, causing endothelial dysfunction, and vasoconstriction, while also promoting mitochondrial dysfunction in cardiomyocytes. ICIs induce myocarditis through T-cell infiltration and cytokine-mediated inflammation. (Tyrosine kinase inhibitors- TKIs, immune checkpoint inhibitors- ICIs, nitric oxide- NO, electron transport chain- ETC, reactive oxygen species- ROS)

Chemotherapy-induced cardiotoxicity can cause an acute or delayed onset cardiac dysfunction, with a higher incidence observed in patients receiving high doses of anthracyclines or those with pre-existing CVD [99, 100]. Chemotherapeutic agents contribute to cardiotoxicity via oxidative stress, mitochondrial dysfunction, and DNA damage. These processes often result in cardiomyocyte loss, leading to pathological remodelling of the myocardium, resulting in arrhythmia and cardiomyopathy [101–103]. A summary of different cancer therapies and CTR-CVT has been illustrated in Fig. 1.

### Anthracycline-mediated cardiotoxicity mechanisms

Anthracycline exposure causes two significant events that lead to cardiomyocyte injury/death (Fig. 1). First, anthracyclines bind to cardiomyocytes, and their quinone moiety is reduced to a semiquinone radical, which oxidises in the presence of molecular oxygen to reform its parent state. This redox cycling in anthracycline-iron complexes generates ROS. Second, the formation of the topoisomerase-anthracycline complex prevents the resealing of phosphate bonds in DNA, causing DNA damage [18–20, 104]. This activates pro-apoptotic and pro-inflammatory cytokines, which degrade mitochondrial structure and function in cardiomyocytes [105]. DNA damage activates extracellular signal-regulated kinase1/2 (ERK1/2), enhances p53 phosphorylation, a crucial cell-cycle regulator, and upregulates pro-apoptotic genes such as *PUMA* and *NOXA* [106, 107]. *PUMA* activates *BAX*/*BAK,* which disrupts mitochondrial integrity, leading to the release of pro-apoptotic factors like cytochrome c, *SMAC*, and *AIF*, which activate caspases and initiate cell death [108]. Alternatively, DOX induce apoptosis in cardiomyocytes by activating NF-kB, which upregulates *PUMA*, highlighting the dependency of *PUMA* on both the p53 and NF-kB pathways [107]. External ligand binding of Fas-L and tumour necrosis factor to their respective receptors stimulates cell death signalling, contributing to cardiomyocyte damage/injury [109].

The cardiotoxic effect of anthracycline on endothelial dysfunction disrupts vascular health and leads to the development of conditions majorly into atherosclerosis, cardiomyopathy, vasoconstriction [110, 111]. This endothelial dysfunction is driven by the production of ROS which impairs blood flow, diminishes NO availability, and accelerates the progression of build-up plaque in the walls of arteries [112]. Several molecular (endothelin-1) and genetic factors (genetic polymorphism of endothelial nitric oxide synthase, NADPH oxidase) play a crucial role in the development of vascular toxicity and endothelial damage, eventually leading to HF [110]. Anthracyclines downregulate antioxidant defences like *SVCT*-*2* and *GPX* [113]. Mitochondrial dysfunction is also caused by iron accumulation via upregulation of transferrin receptor expression, iron regulatory proteins, and downregulation of *ABCB8* [114, 115]. This reduces peroxisome proliferated-activated receptor (PPAR) expression, which affects Acetyl CoA carboxylase (ACC), leading to excessive malonyl-CoA production and ATP consumption [116, 117]. Anthracyclines promote mitochondrial fission and inhibit fusion. This is due to enhanced mitophagy resulting from the *PINK1*/Parkin pathway activation via increased *Drp*-*1* levels and reduced *MFN1/2* and *OPA1* expression [118].

### Targeted therapy-mediated cardiotoxicity mechanisms

ERBB2 is a receptor protein known for its significant role in cell signalling, especially in response to Neuregulin (NRG-1) within cardiomyocytes. NRG-1 leads to the dimerization of ERBB2/4, unlocking the cell survival signalling pathway under adaptation and stress conditions in cardiomyocytes. The NRG-1/ERBB2 signalling is responsible for cardiac tissue development and proliferation [32, 119]. However, trastuzumab binding to HER2/ERBB2 receptors inhibits NRG-1-mediated ERBB2/4 dimerization. This triggers NRG-1 release from the cardiac endothelial cells, leading to disrupted cardiomyocytes through oxidative stress and pro-apoptotic signals [33]. Increased ROS accumulation and oxidative stress have been observed in patients with trastuzumab in combination with anthracycline, resulting in cardiac dysfunction and the development of CHF and left ventricular dysfunction [120].

Cardiomyocyte damage from TKIs such as bevacizumab arises from mechanisms involving cardiomyocyte and endothelial dysfunction [37]. They suppress endothelial nitric oxide (NO) synthase activity and reduce NO levels, contributing to oxidative stress and endothelial cell apoptosis [121]. Chronic endothelial dysfunction also leads to microvascular rarefaction mediated hypertension and myocardial strain. It also reduces myocardial perfusion and increases susceptibility to ischemic injury [38, 122]. By affecting cardiomyocyte survival pathways such as PI3 K/Akt signalling, vascular endothelial growth factor (VEGF) inhibition accelerates mitochondrial dysfunction and apoptosis within cardiac cells [123]. SOR causes cardiomyocyte death via the accumulation of lipid-mediated ROS in the endoplasmic reticulum [124]. Unlike anthracyclines, ICIs cause immune mediated cardiotoxicity, which typically manifests shortly after the first two cycles of treatment [92]. This severe myocarditis is linked to a surge of T cells infiltrating the heart, due to increased levels of pro-inflammatory cytokines, contributing to cardiac damage and direct myocardial toxicity over time through oxidative stress and mitochondrial disruption [41].

## Radiotherapy

External beam radiotherapy is important for treating 60% of cancer patients. During radiotherapy of the cancers of breast, head and neck, oesophagus, lung, and Hodgkin lymphoma, the heart is exposed to radiation, and the dose varies depending on the technique and individual patient’s anatomy. A major consequence of radiotherapy generally observed in patients is late cardiotoxicity [45, 125]. The clinical presentation comprises coronary artery atherosclerosis, valvular disease, pericarditis, cardiomyopathy, and conduction defects. The estimated prevalence of radiotherapy-induced cardiomyopathy is > 10%, with an increased incidence of heart failure compared to that of the general population [45]. Cancer survivors who underwent mediastinal irradiation showed pathological myocardial remodelling leading to a DCM-mediated systolic dysfunction or restrictive cardiomyopathy-mediated diastolic dysfunction [126, 127].

The risk of radiotherapy-induced cardiotoxicity is influenced by the type of cancer, dose, patient’s age, medical history, smoking status and high blood pressure [44]. Women with left-sided BC radiotherapy have a higher chance of developing ischemic heart disease, especially under combination therapy [128]. Over the decades, advances in radiotherapy techniques have reduced the mean heart dose to 4.7 Gy by the 1990 s to 2.6 Gy in 2006 [129]. Although the mean heart dose is a suitable parameter, it does not imply that lowering the dose will not have any effect on the cardiac system, especially when the areas, like the left anterior descending artery and left ventricle (LV), receive high doses  [130].

The Pediatric Normal Tissue Effects in the Clinic (PENTEC) initiative found that higher cardiac radiation in combination with anthracycline doses significantly increases the risk of late cardiac disease in childhood cancer survivors. A mean heart dose < 10 Gy at standard fractionation poses a low 30-year risk without anthracycline exposure [131].

Recent advancements in radiotherapy focus on improving precision, reducing toxicity and enhancing outcomes; however, cardiotoxicity remains a predominant factor with advancements like hypofractionation. Damaging effects on the heart can be seen after doses as low as 2 Gy, and there is no apparent “safe” dose [132]. Doses of 40 Gy, 42.5 Gy, and 39 Gy have equivalent efficiency to the regular fractionation regime of 50 Gy, and it is shown to cause less cardiac damage [133]. A study of 5,58,871 subjects recorded over 20 years revealed that women with left-side BC receiving radiotherapy had a 90% higher risk of cardiac death compared to right-sided BC patients [134].

### Radiation therapy-induced cardiotoxicity mechanisms

**Fig. 2:**
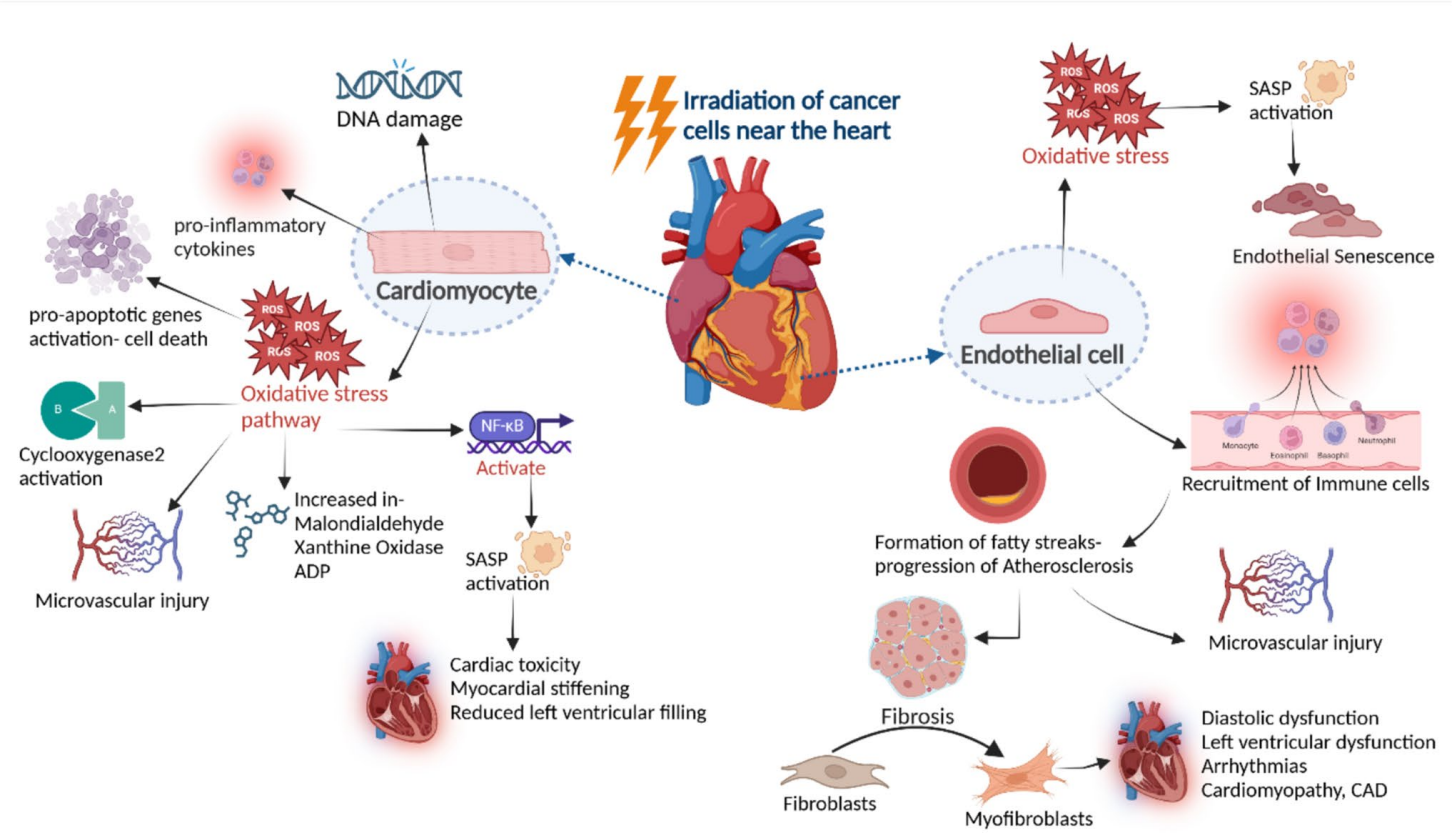
The diagram illustrates the mechanisms of radiation-induced cardiac toxicity. Radiation-induced cardiomyocyte damage occurs due to oxidative stress, and DNA damage. Oxidative stress triggers pro-inflammatory cytokines, pro-apoptotic gene activation, increased malondialdehyde and xanthine oxidase, and cyclooxygenase-2 activation, resulting in microvascular injury, cell death, myocardial stiffening, and reduced left ventricular filling. In endothelial cells, oxidative stress promotes senescence, SASP activation, and immune cell recruitment, contributing to fibrosis through fibroblast activation, fatty streak formation, and atherosclerosis progression. These processes result in microvascular damage, diastolic dysfunction, arrhythmias, cardiomyopathy, and CAD, highlighting the interconnected pathways in radiation-induced cardiac toxicity. (reactive oxygen species- ROS, senescence-associated secretory phenotype- SASP, coronary artery disease (CAD)

Proposed pathogenic mechanisms of radiation-induced CVD include endothelial cell damage with accelerated atherosclerosis, pro-thrombotic alterations in the coagulation pathway, inflammation, and fibrosis of the myocardial, pericardial, valvular, and conduction tissues [135]. A summary of the mechanisms of radiation induced cardiotoxicity is illustrated in Fig. 2. Radiation-induced oxidative stress in cardiomyocytes causes increased levels of malondialdehyde, xanthine oxidase, and adenosine deaminase, causing microvascular injury, reduced myocardial capillary density, and decreased oxygen [136]. Patients with pre-existing CVD develop cardiotoxicity due to microvascular injury, leading to rapid pericardial effusion [137].

Radiation induces inflammation through cyclooxygenase-2 (COX-2), an enzyme that converts prostaglandin from arachidonic acid [42]. After radiation exposure, damage response signals are activated from nuclear DNA and mitochondria via the upregulation of ROS and genes like *ATM*, *p53*, *p16*, and *p21*. ROS production activates NF-κB that transcribes senescence-associated secretory phenotype (SASP), leading to senescence. Activating these pathways damages cells in and around the cardiomyocytes causing chronic inflammation and disruption of cardiac tissue structure [43, 138].

A similar mechanism is seen in endothelial cells, initiating endothelial senescence and inflammation. Endothelial dysfunction begins with the accumulation of apolipoprotein B, a low-density lipoprotein in the arteries, which triggers the recruitment of monocytes and macrophages into the endothelium and then transforms into macrophages and develops a fatty streak in the inner lining of the endothelium. This marks the beginning of atherosclerosis, leading to myocardial infarction and fibrosis due to ischaemia and cell death [43, 139]. The blood vessel elasticity is impaired due to the accumulation of extracellular matrix components. Several inflammatory molecules and cytokines contribute to this process, including tumour necrosis factor, interleukin (IL)−1, IL-6, and platelet-derived growth factors. Further, monocyte chemotactic factor and transforming growth factor are also known to play a role in this process [140]. Myocardial metabolic alterations may play an important role in the pathogenesis of radiation-induced cardiotoxicity. Radiation leads to bioenergetics failure in cardiomyocytes and metabolic alterations such as a shift from fatty acid oxidation to glycolysis, as observed in ischemic heart failure [141].

Cardiomyocyte loss secondary to radiation leads to myocardial replacement fibrosis, which causes pathological remodelling of the heart and changes its structure and function. The formation of myofibroblasts from cardiac fibroblasts signals the onset of cardiac fibrosis, with several signalling pathways, including PI3 K/AKT, TGF-β/Smad3, and MAP/ERK2 playing a role [142]. Interstitial fibrosis of the myocardium can cause cardiomyopathies, arrhythmias, coronary artery disease (CAD) and valvulopathies. Cardiomyopathies are progressive disorders which lead to heart failure, and arrhythmias can cause sudden cardiac death. Pericardial fibrosis can lead to thickening of the pericardium and cause abnormalities in cardiac function [143, 144].

## Cardioprotective strategies

The growing understanding of the cardiotoxic mechanisms of various cancer treatment modalities has resulted in several treatment strategies which ameliorate cardiotoxicity [102]. A list of studies that established these cardioprotective treatments is summarised in Table 2.

**Table 2:** A table summarising the cardioprotective strategies, their mechanisms, and key findings from clinical studies. (renin-angiotensin system, angiotensin-converting enzymes-ACE, angiotensin-receptor blocker- ARBs, intensity-modulated radiation therapy-IMRT, Deep inspiration breath-hold- DIBH, left ventricular ejection fraction- LVEF, Doxorubicin- DOX, Left ventricle-LV)

Cardioprotective Strategy	Mechanism/Effect	Key findings	References
**Pharmacological approaches**			
β-blockers	Treat hypertension by slowing heart rate and relaxing blood vessels	Mixed results for cardiotoxicity in cancer patients; Carvedilol and nebivolol improved diastolic dysfunction	[145, 146]
RAS inhibitors (ACE inhibitors and ARBs)	Control hypertension, oxidative stress, and cardiac fibrosis	Reduce hospitalization rates more effectively than β-blockers but may cause complications like hyperkalemia and angioedema in renal disease patients	[147–150]
Dexrazoxane	Reduces cardiotoxicity by inducing DNA repair mechanisms and inhibiting mitochondrial damage and ROS formation	Effective in patients receiving high-dose anthracyclines	[151, 152]
Combination therapy	Synergistic effects e.g., β-blockers and RAS inhibitors	Improves outcomes such as reduced left ventricular hypertrophy and cardiac dysfunction; PRADA trial showed early benefits with candesartan and metoprolol, but long-term benefits were modest. MANTICORE-101 trial showed no benefit in LV remodelling	[153–156]
Statins	Lower cholesterol, reduce oxidative stress, and inflammation	No significant cardioprotective properties observed in PREVENT, SPARE-HF, and STOP-CA trials; recommended for high-risk patients per ESC guidelines	[157–159]
**Radiation therapy advances**			
Intensity-modulated radiation therapy (IMRT) and proton therapy	Precisely target cancer and spare healthy tissue	Minimize cardiac exposure and reduce cardiotoxicity	[138]
FLASH radiotherapy	Delivers ultra-high doses in a single fraction, reducing tissue damage	Experimental stage with promising results in reducing cardiotoxicity	[160]
Deep inspiration breath-hold (DIBH)	Inflates lungs to shift the heart away from the treatment area	Minimizes cardiac radiation risk, especially in breast and lung cancer treatments	[161, 162]
**Natural compounds**			
Chia seed oil	Reduces oxidative stress, enhances antioxidant enzymes	Cardioprotective against DOX in rats	[163]
Ajwa date nanopreparation	Increases antioxidant capacity, suppresses inflammation, reduces ischemia	Cardioprotective against DOX-induced cardiotoxicity	[164–166]
Curcumin	Potent antioxidant derived from *Curcuma longa*	Mitigates DOX-induced cardiotoxicity; improves cardiac function	[167]
Flavonoids	Antioxidant, cardioprotective, and anti-tumor properties	Mechanisms need further exploration	[168]
Cannabidiol	Antioxidant and anti-inflammatory properties	Evaluated in mouse models of anthracycline-induced cardiomyopathy	[169, 170]
**Lifestyle modification and Exercise**			
Exercise	Enhances cardiovascular health, restores cardiomyocyte homeostasis	Reduced mortality in cancer patients by 48%; improves VO2 peak in BC patients, but LVEF impact remains unclear	[171, 172]
Lifestyle changes	Smoking cessation, reduced alcohol intake, healthy weight maintenance	Crucial for prevention in cardiovascular toxicity, especially in hepatocellular carcinoma patients	
Tailored interventions (e.g., yoga, qigong)	Improves quality of life	Requires more research to confirm cardioprotective mechanisms	[173]

Using fewer cardiotoxic chemotherapeutics or lower drug dosages while maintaining treatment effectiveness has been the most commonly accepted cardioprotective strategy in chemotherapy [102, 135]. Pharmacological approaches such as angiotensin converting enzyme (ACE) inhibitors, angiotensin-receptor blockers (ARBs), β-blockers, dexrazoxane, and statins confer cardioprotective effects by maintaining normal heart function and preventing the development of cardiotoxicity. β-blockers treat hypertension by slowing the heart rate and relaxing blood vessels. Using β-blockers as a standalone treatment for cancer patients experiencing cardiotoxicity has yielded mixed results. In one study, carvedilol and nebivolol improved diastolic dysfunction in anthracycline-treated patients [145]. Another study, using echocardiogram strain-guided imaging, found that carvedilol had no significant advantage as a cardioprotective drug in terms of heart function or CTR-CVD prevention. However, the study also stated that cardiac strain changes alone may not warrant immediate intervention with cardioprotective therapy, especially in patients at low risk of cardiac dysfunction [146]. Older patients may experience fatigue, bradycardia, and exercise intolerance when using β-blockers, making personalised treatment necessary [147]. Renin-Angiotensin system (RAS) inhibitor drugs like ACE inhibitors and ARBs such as captopril, enalapril, lisinopril, and valsartan are well-known to confer cardioprotection by controlling hypertension, oxidative stress, and cardiac fibrosis [148–150]. Comparative trials show that RAS inhibitors reduce hospitalisation rates more effectively than β-blockers, although the latter fares better in arrhythmia prevention [174]. Patients with a history of renal disease do not tolerate this treatment as it can cause significant complications like hyperkalemia and angioedema [147]. Dexrazoxane helps reduce cardiotoxicity by inducing DNA repair mechanisms, inhibiting mitochondrial damage and ROS formation in patients of all ages receiving high-dose anthracycline treatment [151, 152].

A recent study found that combination therapy provides better cardioprotective outcomes than standalone drug treatments, particularly in reducing mortality in heart failure patients; however, the treatment must be tailored specifically to the patient’s needs [152]. Combining β-blockers and RAS inhibitors can reduce left ventricular hypertrophy, diastolic, and systolic dysfunction. However, long-term outcomes are determined by specific heart failure subtypes and aetiologies [153]. In the PRADA trial, metoprolol was used in combination with candesartan to assess the cardioprotective effects on BC patients treated with adjuvant therapy. Cardiotoxicity causes acute myocardial damage, which can be measured using cardiac troponin biomarkers [175]. Overall outcomes were measured as LVEF preservation and cardiac troponin readings. Early measurements in the study showed metoprolol did not significantly impact LVEF but attenuated the increase in cardiac troponin, while candesartan treatment showed LVEF preservation but no effect on cardiac troponin levels [154]. Extended follow-up after 2 years revealed a slight decrease in LVEF but no significant long-term preservation of LVEF with candesartan treatment; however, there was a modest reduction in left ventricular end-diastolic volume and preserved global longitudinal strain. These findings suggest that while candesartan provides short-term benefits, a universal cardioprotective approach during adjuvant therapy may not be necessary for most patients without pre-existing CVD, highlighting the importance of individualised cardiac risk assessment [155]. The MANTICORE 101- trial was conducted in HER2-positive BC patients to study the efficacy of the combination of ACE inhibitors and β-blockers in preventing trastuzumab-mediated cardiotoxicity. Perindopril and bisoprolol effectively attenuated LVEF decline; however, neither prevented trastuzumab-mediated LV remodelling, which was the primary outcome [156].

Drug combinations of targeted therapies with taxanes yield better cardiac safety profiles with minimal cardiotoxicity. The CLEOPATRA trial conducted during 2015–2020 demonstrated the cardiac safety of combining pertuzumab, trastuzumab, and docetaxel in treating HER2-positive metastatic BC. This combinatorial treatment achieved an 8-year overall survival rate of 37% [176]. Another study confirmed that treatment with the combination of paclitaxel, trastuzumab, and pertuzumab was cardio-safe [177]. Statins, which lower cholesterol levels by terminating the 3-hydroxy-3-methylglutaryl coenzyme A (HMG CoA) reductase, help reduce oxidative stress and inflammation [157]. These effects make them potentially beneficial in reducing the risk of heart failure during anthracyclines and trastuzumab therapies [158]. Three randomized controlled trials; PREVENT, SPARE-HF, and STOP-CA have investigated the cardioprotective properties of statins. While the evidence indicates that statins do not reduce clinical heart failure rates, the current European Society of Cardiology (ESC) guideline recommends their use for patients at high or very high risk [159].

In radiotherapy, advanced radiation techniques, such as intensity-modulated radiation therapy (IMRT), and proton therapy, can target cancer more precisely and spare healthy tissue [138]. Charged particle therapy, using protons and heavy ions, minimises damage due to the Bragg peak and higher relative biological effectiveness compared to conventional radiation. FLASH radiotherapy delivers ultra-high doses in a single fraction and shows reduced tissue damage through rapid oxygen depletion, although clinical application remains experimental [160]. The breath-holding technique, particularly deep inspiration breath-hold (DIBH), is widely used to reduce cardiac exposure. By inflating the lungs, the heart is shifted away from the treatment area, minimising radiation risk to the heart, particularly in breast and lung cancer treatments [161, 162]. Several studies concluded that DIBH significantly reduces the negative impact of radiation doses to critical organs like the heart and left anterior descending artery [178–180]. S. Tanguturi’s study indicated that the effectiveness of DIBH varied across patients, with better outcomes seen in younger patients with a higher BMI and larger changes in lung volume during inspiration [181]. Despite its beneficial aspects, in a comparative study between DIBH and IMPT, DIBH was not feasible for some patients due to long treatment duration and age, highlighting its limitation in real-world applicability [182]. A study over 3 years compared Volumetric modulated arc therapy (VMAT) and helical tomography (HT) in left BC patients undergoing radiotherapy found no significant deterioration in cardiac function, as assessed by LVEF. The results suggested both VMAT and HT can effectively limit cardiac exposure without causing noticeable damage [183]. Several studies have evaluated the impact of radiation on the cardiac conduction system, the sinoatrial node (SA) and atrioventricular node (AV) nodes. P. Loap et al., demonstrated that both these nodes are exposed during breast irradiation with VMAT, while Intensity-modulated proton therapy (IMPT) offers a potential dosimetric benefit as it delivers virtually no dose to SA and AV nodes [184].

Natural compounds are reliable sources of cardioprotection. Chia seed oil, rich in omega-3 fatty acids, is an effective chemoprotective agent against DOX in rats. It reduces oxidative stress, enhances superoxide dismutase and catalase, and neutralises free radicals generated by DOX [163]. Nanopreparation of ajwa dates showed cardioprotection against DOX-induced cardiotoxicity by increasing cardiac antioxidant capacity, suppressing inflammatory pathways mediated by cytokines and alleviating ischemia by upregulating glutathione and reducing malondialdehyde levels [164–166]. Curcumin, derived from *Curcuma longa*, is a potent antioxidant that mitigates DOX-induced cardiotoxicity. It reduces oxidative stress and improves cardiac function, promising an adjunctive strategy for cancer therapies [167]. Certain flavonoids have shown both cardioprotective and anti-tumour properties; however, their mechanisms are still to be discovered [167]. While traditional strategies help mitigate cardiotoxicity, a recent alternative to traditional Chinese medicine has gained attention for its potential in cardioprotection. Particularly, flavonoids, having both anti-protective and anti-tumour properties highlights the need to explore their mechanism in alleviating cardiotoxicity [168]. Non-psychotropic constituent of *Cannabis sativa*, cannabidiol, has antioxidant and anti-inflammatory properties, as was evaluated in mouse models of anthracycline-induced cardiomyopathy [169, 170].

**Fig. 3:**
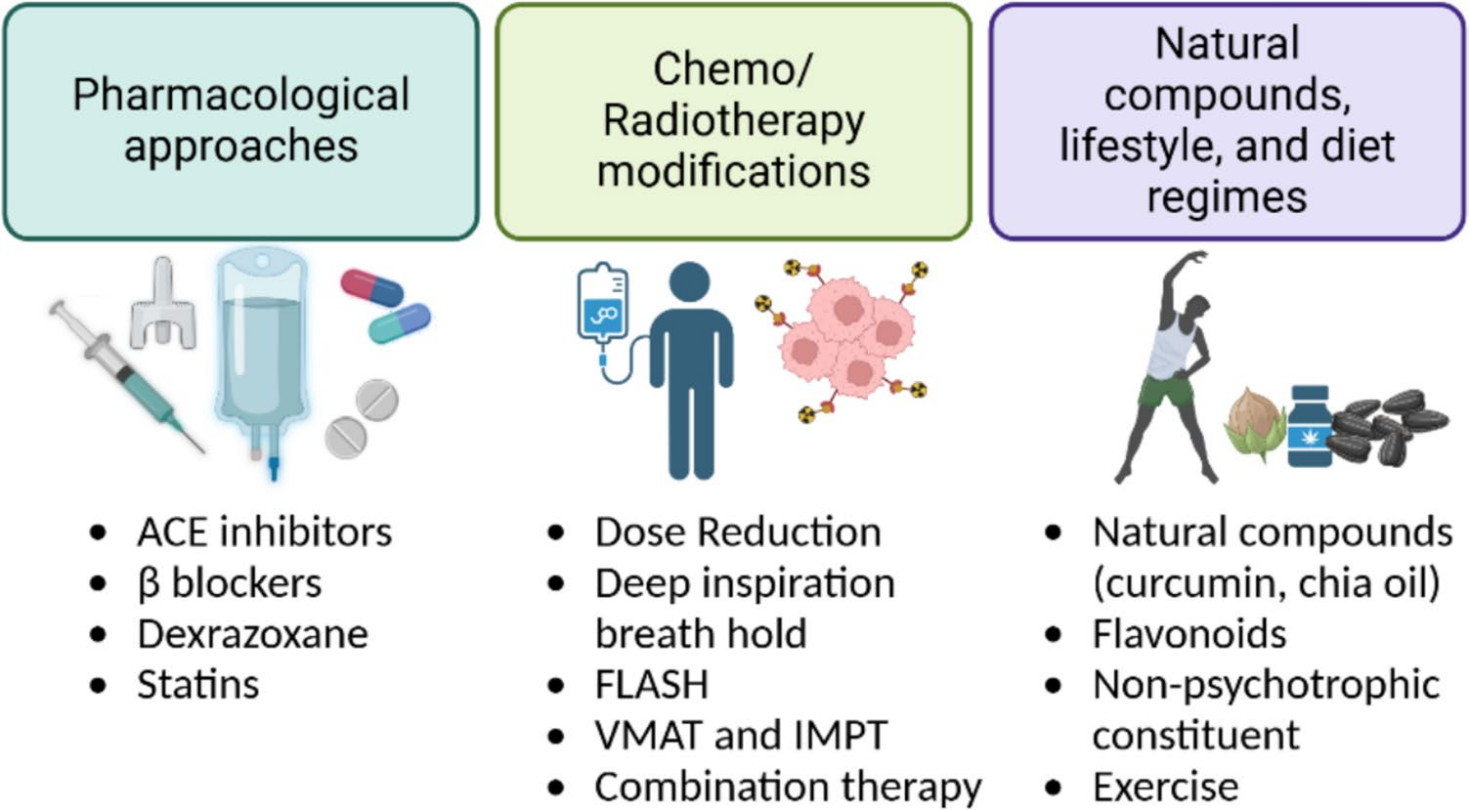
Strategies to mitigate cardiotoxicity in cancer treatments

Lifestyle modifications and exercise can improve cardiovascular health and reduce the impact of cancer treatment on the heart. Cancer patients develop deteriorated muscle strength due to radio/chemo drug exposure. Studies have shown that physical strength restores cardiomyocyte homeostasis and the heart's tolerance to chemotherapeutic agents [185]. Recent findings of a systematic review and meta-analysis of randomised controlled trials found exercise reduced cardiac mortality risk in cancer patients by 48%, although limitations included a small sample number (n = 3), moderate quality, and unclear definitions of cardiac mortality [171]. Aerobic exercise improved VO2 peak in BC patients, but its direct impact on LVEF remains uncertain due to small sample sizes and protocol variability, emphasising the need for high-quality trials to confirm its role in preventing cardiotoxicity [172]. Additionally, tailored interventions such as psychological treatments, including yoga, exercise, and qigong/Tai Chi have been proven to enhance the quality of life, though more research is needed to assess their cardioprotective mechanism [173]. A summary of different cardioprotective strategies in cancer therapy is illustrated in Fig. 3.

## Conclusion

Cancer treatment-induced cardiotoxicity is a notable concern in managing cancer patients and can significantly impact the quality of life of cancer survivors. The clinical manifestation of cardiotoxicity varies; it is primarily progressive and depends on the type and duration of cancer therapy. The clinical manifestations of CTR-CVT can range from acute symptoms to chronic heart dysfunction, with late-onset cardiotoxicity presenting significant challenges for cancer survivors. Several in vitro and preclinical models have advanced our study of the effects of cardiotoxicity of these cancer treatment modalities, but they have certain limitations. In vitro cultures, such as 2D cardiomyocytes lack replicability of in vivo cardiac environments. HiPSC-CMs are emerging as a transformative tool for pre-clinical cardiotoxicity assessments; however, much progress must be made in translating this innovation into clinical reality. Their current challenges, such as variability in reprogramming quality, epigenetic memory affecting differentiation efficiency, and unpredictable reactions during cell reprogramming, highlight the unmet need for refined techniques. Preclinical animal model is hindered by interspecies differences in cardiovascular physiology and drug responses, as well as their inability to replicate the genetic and metabolic diversity of the human population.

The mechanistic insights reveal that chemotherapy, targeted therapy, and radiotherapy exert cardiotoxicity primarily by oxidative stress and cellular damage. Other mechanisms include alterations in apoptosis, epigenetic modifications, and genetic susceptibility leading to cardiomyocyte and endothelial damage. TKIs induce cardiotoxicity by suppressing NO levels necessary for vascular homeostasis. This causes a strain on the myocardium, resulting in vasoconstriction. The loss of cardiomyocytes leads to pathological remodelling of the myocardium, leading to arrhythmias and cardiomyopathies. On the other hand, ICIs cause T-cell infiltration that manifests in myocarditis and acute cardiac injury. The damage to endothelial cells can lead to atherosclerosis and CAD.

To alleviate the significant cardiotoxic effects of cancer therapies, new mitigating strategies are being developed and tested. Cardio-oncology has seen considerable advancements, including multidisciplinary collaborations and the establishment of tailored clinical trials. For example, the PRADA trial evaluated the efficacy of ACE inhibitors and β-blockers in reducing chemotherapy-induced cardiotoxicity, while the CLEOPATRA trial highlighted the cardiac safety of combining targeted therapy with taxanes for HER2-positive BC. These combination therapies have shown synergistic effects on reducing oxidative stress and improving cardiac output, albeit with limited long-term outcomes. Moreover, novel compounds, exercise, and lifestyle modifications show promising results to counter cancer therapy’s toxic cardiac effects. Efforts to develop biomarkers like troponin and advanced imaging techniques hold promise but require further validation for widespread clinical adoption. In radiotherapy, DIBH techniques, and proton therapy offer a potential dosimetric benefit to reduce cardiac toxicity, although limitations like real-life applicability remain. Developing newer biomarkers and imaging techniques for continuous monitoring of clinical symptoms and identifying the early cardiotoxic effects can be helpful in preventing severe toxicities.

Future research should focus on understanding the mechanisms of cancer therapy-induced cardiotoxicity and develop more effective strategies to bridge the gap between mechanistic insights and clinical applications. This would require sustained efforts in interdisciplinary research, innovation, and collaboration from multiple oncology specialties, cardiology, radiation therapy, physiotherapy, and nutrition science. Studies should be conducted to incorporate preventive genetic testing and integrate pre-treatment protocols that could allow for dose adjustments or alternative therapies, potentially reducing adverse outcomes. A foremost focus on developing optimised prevention methods enhanced diagnostic accuracy, and personalised treatments will increase patients'quality of life during and post cancer treatment.

## Data Availability

No datasets were generated or analysed during the current study.

## Abbreviations

HMG-CoA: 3-hydroxy-3-methylglutaryl coenzyme A
ACE: Angiotensin-converting enzymes
ACC: Acetyl CoA carboxylase
ARB: Angiotensin-receptor blockers
AV: Atrioventricular node
BC: Breast Cancer
CTR-CVT: Cancer therapy-related cardiovascular toxicity
CVD: Cardiovascular disease
CAD: Coronary artery disease
CHF: Chronic Heart Failure
CTLA-4: Cytotoxic T lymphocytes associated antigen 4
DIBH: Deep inspiration breath-hold
DPD: Dihydropyrimidine dehydrogenase
DCM: Dilated Cardiomyopathy
DOX: Doxorubicin
ESC: European Society of Cardiology
FU: Fluorouracil
HT: Helical tomography
(hiPSC-CMs): Human induced pluripotent stem cell-derived cardiomyocytes
NRG-1: Neuregulin
NO: Nitric oxide
ICIs: Immune Checkpoint Inhibitors
IMRT: Intensity-modulated radiation therapy
LV: Left ventricle
LVEF: Left Ventricular Ejection Fraction
PPAR: Peroxisome proliferated-activated receptor
RAS: Renin-Angiotensin system
ROS: Reactive oxygen species
SA: Sinoatrial node
SOR: Sorafenib
TKIs: Tyrosine kinase Inhibitors
VEGF: Vascular endothelial growth factor
VMAT: Volumetric modulated arc therapy
